# Quantifying Citrate Surface Ligands on Iron Oxide Nanoparticles with TGA, CHN Analysis, NMR, and RP-HPLC with UV Detection

**DOI:** 10.1021/acs.analchem.5c03024

**Published:** 2025-08-29

**Authors:** Anna Matiushkina, Sarah-Luise Abram, Isabella Tavernaro, Robert Richstein, Michael R. Reithofer, Elina Andresen, Matthias Michaelis, Matthias Koch, Ute Resch-Genger

**Affiliations:** † Division of Biophotonics, 42220 Bundesanstalt für Materialforschung und -prüfung (BAM), Richard-Willstaetter-Straße 11, 12489 Berlin, Germany; ‡ Department of Biology, Chemistry, and Pharmacy, Free University Berlin, Arnimallee 22, 14195 Berlin, Germany; § Institute of Inorganic Chemistry, 27258 University of Vienna, Waehringer Straße 42, 1090 Vienna, Austria; ∇ Institute of Inorganic Chemistry, Faculty of Chemistry, University of Vienna, 1090 Vienna, Austria; ∥ Division Organic Trace and Food Analysis, Bundesanstalt für Materialforschung und -prüfung (BAM), Richard-Willstaetter-Straße 11, 12489 Berlin, Germany

## Abstract

Although citrate is frequently used as a surface ligand for nanomaterials (NMs) such as metal, metal oxide, and lanthanide-based NMs in hydrophilic environments due to its biocompatibility and simple replacement by other more strongly binding ligands in postsynthetic surface modification reactions, its quantification on NM surfaces has rarely been addressed. Here, we present a multimethod approach for citrate quantification on iron oxide nanoparticles (IONPs) broadly applied in the life and material sciences. Methods explored include thermogravimetric (TGA) and elemental (CHN) analysis, providing citrate-nonspecific information on the IONP coating, simple photometry, and citrate-selective reversed-phase high-performance liquid chromatography (RP-HPLC) with absorption (UV) detection and quantitative nuclear magnetic resonance spectroscopy (qNMR). Challenges originating from the strongly absorbing magnetic NM and paramagnetic iron species interfering with optical and NMR methods were overcome by suitable sample preparation workflows. Our multimethod approach to citrate quantification highlights the advantages of combining specific and unspecific methods for characterizing NM surface chemistry and method cross-validation. It also demonstrates that chemically nonselective measurements can favor an overestimation of the amount of a specific surface ligand by signal contributions from molecules remaining on the NM surface, e.g., from particle synthesis, such as initially employed ligands and/or surfactants. Our results emphasize the potential of underexplored selective RP-HPLC for quantifying ligands on NMs, which does not require a multistep sample preparation workflow such as qNMR for many NMs and provides a higher sensitivity. These findings can pave the road to future applications of versatile HPLC methods in NM characterization.

## Graphical abstract

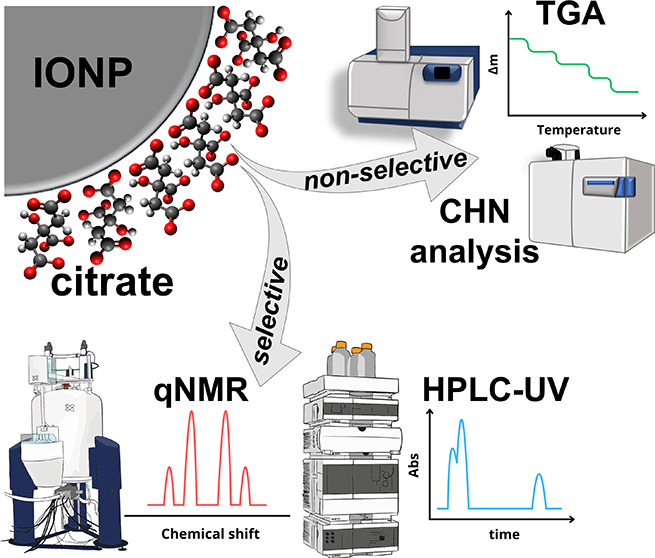

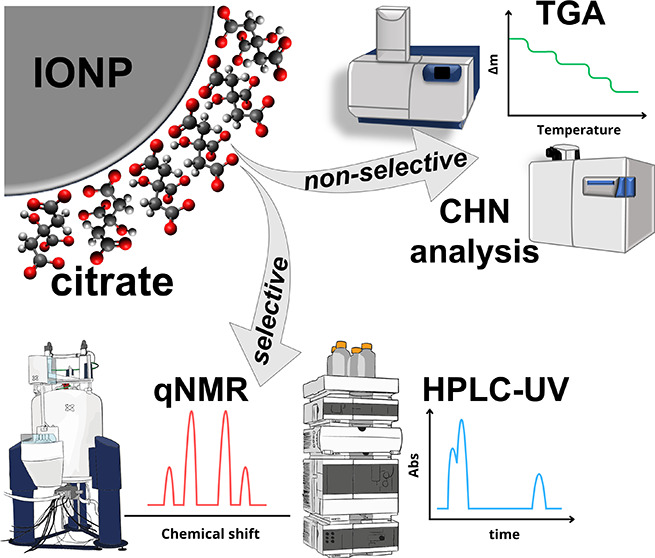

## Introduction

Engineered inorganic, organic, and hybrid nanomaterials (NMs), which commonly present core or core/shell nanostructures stabilized with covalently or coordinatively bound surface ligands, are meanwhile widely used in the material and life sciences, e.g., in nanomedicine,^1^ medical diagnostics,^2^ for energy conversion and storage,^3^ optoelectronics,^4^ and catalysis.^5^ Application-relevant NM features such as optical and magnetic properties are largely determined by size, morphology, composition, and crystal phase, while the surface chemistry controls the colloidal stability, dispersibility, processability, and NM interaction with the environment and biological species, and hence exposure and potential toxicity.^6,7^ This highlights the importance of methods for the controlled surface functionalization of NMs^8,9^ and analytical methods to quantify ligands and functional groups (FGs) on NM surfaces^6,10,11^ as well as test and reference materials for method validation.^12^ The latter is also relevant for property-application and property-safety relationships to enable NM grouping, and to ease the development of the next generation of safe and sustainable by design (SSbD) NMs.^7^

Analytical methods to determine and quantify surface FGs and ligands include quantitative nuclear magnetic resonance (qNMR) techniques,^13^ X-ray photoelectron spectroscopy (XPS),^14^ vibrational spectroscopy, e.g., Fourier-transform infrared (FTIR) and Raman spectroscopy,^15^ thermogravimetric analysis (TGA),^16^ inductively coupled plasma mass spectrometry or optical emission spectroscopy (ICP-MS or ICP-OES),^11,17^ and electrochemical titration techniques such as conductometry and potentiometry.^6,18^ Chemo-selective methods such as solid state or solution qNMR can quantify ligands either on NMs or in solution after NM dissolution,^13,19,20^ while FTIR or Raman yield only semiquantitative results.^6,11^ Other methods provide less specific information such as surface coating mass loss like TGA, unless combined with mass spectrometry or FTIR, yield only the total amount of (de)­protonable FGs such as conductometry, or are solely suitable for ligands containing heteroatoms, such as sulfur, like ICP-MS and ICP-OES.^6^ Also, simple optical assays are frequently employed, which require a chemical reaction or an electrostatic interaction of the FGs and the signal-generating reporter.^18,21^ Such assays yield the assay-specific amount of reporter-accessible FGs, which depends on reporter size and charge.^13^ The applicability of all these analytical methods for NM surface analysis depends on NM composition and surface ligand(s) and the NM-ligand bonding interactions. This also determines mandatory sample preparation steps prior to ligand analysis, such as NM removal from dispersion or NM dissolution, and can affect the accuracy of the measurements.^22^

One of the most frequently utilized surface ligand for stabilizing metal, metal oxide, and lanthanide nanoparticles (NPs) in hydrophilic environments^23^ is citrate due to its biocompatibility and simple replacement by other more strongly binding ligands in postsynthetic surface modification reactions.^9,24^ However, the quantification of citrate on NM surfaces has rarely been addressed, although it is a frequent analyte in medical and food analysis.^25^ Examples present the determination of citrate on gold, silver, and iron oxide NPs (IONPs) using TGA, elemental (CHN) analysis, and FTIR.^26^ The frequent use of citrate ligands encouraged us to develop and compare different methods for citrate quantification on NMs representatively for IONPs broadly applied in the life sciences, e.g., for magnetic resonance imaging (MRI), magnetic separation, and magnetic hyperthermia,^27^ with a focus on assessing the potential of versatile high-performance liquid chromatography (HPLC) methods still underexplored for quantifying surface ligands on NMs. Methods explored, varying in citrate selectivity, include photometry, TGA, CHN analysis, as well as reversed-phase high-performance liquid chromatography (RP-HPLC) with absorption (UV) detection and solution qNMR. Challenges originating from the strongly absorbing and magnetic IONPs and their constituting paramagnetic iron ions interfering with optical and NMR methods were tackled by specific sample preparation workflows. Our multimethod approach to citrate quantification, which demonstrates the applicability of RP-HPLC for NM ligand analysis and solution qNMR for magnetic NMs, highlights the importance of combining selective and unspecific methods for NM surface analysis, easing method validation by cross-comparison, and shows limitations of ligand-unspecific measurements. Our results can pave the road to a frequent usage of versatile HPLC methods in NM characterization workflows.

## Experimental Section

### Synthesis of Oleate-Capped IONPs and Ligand Exchange for Citrate

The IONPs were synthesized following the procedure from Park et al.,^28^ using a thermal decomposition of iron oleate in 1-octadecene with oleic acid at 320 °C under an argon flow. To exchange oleate (OA) for citrate (CA) ligands, the resulting oleate-capped IONPs (IONPs-OA) were redispersed in 1,2-dichlorobenzene, mixed with citric acid (CitAc; mass ratio IONPs:CitAc of 1:1) in *N,N*-dimethylformamide (DMF), and stirred at 100 °C for 24 h.^24^ The resulting citrate-modified IONPs (IONPs-CA) were purified by centrifugation using diethyl ether, acetone, and Milli-Q water for washing, redispersed in aqueous sodium hydroxide solution (pH 9.8), followed by filtering with a 0.2 μm syringe filter (CHROMAFIL Xtra H-PTFE, Macherey-Nagel). IONPs-CA samples with a mass concentration of 9.2 g/L were stored in water at a pH of 9.8 at room temperature (r.t., 23 °C). A list of the materials used is given in the Supporting Information (SI).

### Particle Characterization

The particle characterization was done by dynamic light scattering (DLS) yielding the number-based hydrodynamic diameter via cumulants fitting, as well as by zeta potential measurements at pH 9.2 using a Zetasizer Nano ZS (Malvern Panalytical), equipped with a 633 nm laser. The measurements were conducted at 25 °C using NP dispersions with concentrations of about 1 and 2 g/L. Transmission electron microscopy (TEM) images were obtained on a Talos F200S microscope (Thermo Fisher Scientific) and analyzed using the software ImageJ (Version 1.54g), evaluating 300 and 500 particles for IONPs-OA and IONPs-CA, respectively. The IONP iron content was determined by ICP-OES using a SPECTRO Arcos-EOP (Model: FHX, 76004553) spectrometer (SPECTRO Analytical Instruments) and iron ICP standard solution (SRM from NIST, Sigma-Aldrich).

### Citrate Quantification

Photometric measurements were performed with a Cary 5000 spectrophotometer (Agilent) using 2 mm quartz cuvettes. TGA measurements of dried samples were done with a Hitachi STA 7200 setup with an AS3 Sample Charger under argon atmosphere at heating rates of 10 °C/min for IONPs-CA and 20 °C/min for IONPs-OA and sodium citrate dihydrate, and CHN analysis was performed with a PerkinElmer 2400 Series II analyzer. For the RP-HPLC studies, a 1260 Infinity system (Agilent Technologies) equipped with a diode array detector (DAD) was employed with an Eurospher II 100–5 C18 (250 × 4.6 mm) column. The mobile phase consisted of a phosphate buffer (pH 2.9 ± 0.1)-methanol mixture (97.5:2.5), the flow rate was 1 mL/min, and the detection wavelength set to 210 nm. Solution qNMR measurements done at 25 °C with a Bruker BioSpin AV III 600 spectrometer operating at 600.25 MHz for ^1^H detection, using the maleic acid signal (6.57–6.65 ppm, 2H) as internal reference for quantifying the citric acid signals (3.03–3.10 ppm, 2H; 3.21–3.28 ppm, 2H).

### Calculation of the Citrate Concentration

All calculations were done for maghemite (γ-Fe_2_O_3_), using a density ρ of 4.9 g/cm^3^, and a molar mass of citrate of 189 g/mol. The reported uncertainties are standard deviations (SDs) derived from measurements commonly performed in triplicate.

The detailed experimental procedures and calculations are given in the SI.

## Results and Discussion

An overview of the multimethod approach utilized for citrate quantification on IONPs, including IONP characterization (top), sample preparation (middle), and ligand quantification methods (bottom), is presented in Figure 1. To quantify the (average) number of citrate ligand molecules per IONP or ligand density (ligands per NP surface area), i.) the NP surface and hence IONP size and shape, ii.) the NP number concentration, and iii.) the total amount of ligand must be determined. IONP size and shape were characterized by TEM. The absence of IONP aggregation was ensured by DLS measurements as a prerequisite for the calculation of the IONP number concentration from ICP-OES measurements of iron and TEM size, assuming an IONP chemical composition of Fe_2_O_3_. The total amount of organic substances on the IONPs was derived from TGA measuring the mass loss during successive heating.^29^ However, the degradation temperatures are not chemically selective. To quantify a specific ligand, its characteristic ligand properties need to be exploited, such as optical properties or its NMR spectrum. For NM dispersions, a photometric ligand quantification can be hampered by the presence of scattering and/or strongly absorbing NPs such as IONPs, or for dissolved NMs, optical interferences from the NM constituting material or its derivatives.^30,31^ NMR measurements require the absence of (para)­magnetic species.^32^ We first assessed a conventional TGA method and a direct photometric approach, as employed before for quantifying citrate in aqueous solution,^33^ followed by a novel RP-HPLC method with UV detection to separate possibly interfering species prior to photometric citrate quantification. The reliability and accuracy of the results was validated by chemo-selective solution qNMR used before for NM surface analysis, e.g., for aminated silica NPs,^16,20^ and nonselective CHN analysis. Solution qNMR measurements require the quantitative removal of magnetic IONPs and paramagnetic iron ions, and hence a multistep sample preparation workflow.

**Figure 1:**
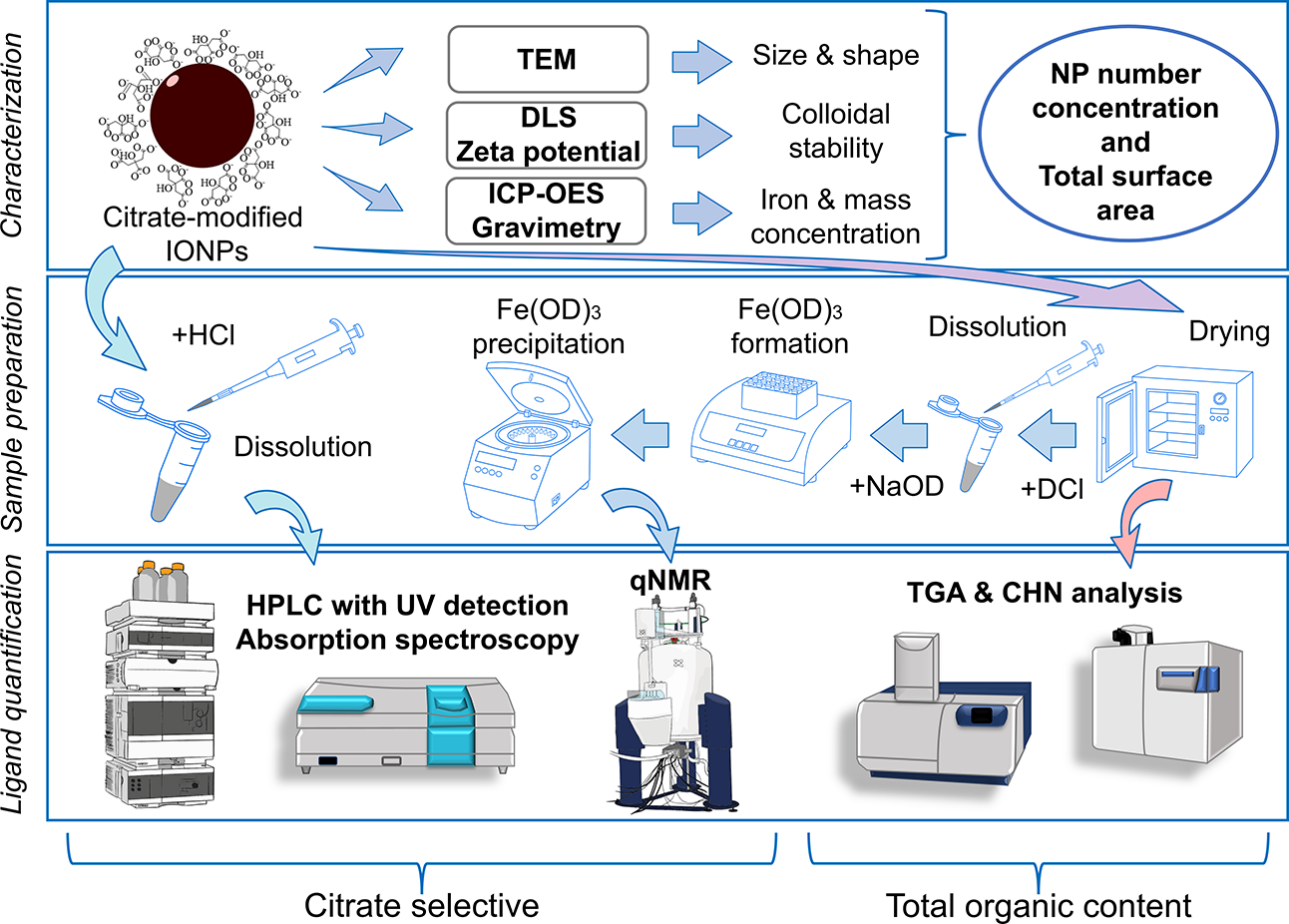
Schematic overview of the multimethod approach to determine citrate in IONPs-CA samples with the methods utilized for (i) particle characterization (top), (ii) sample preparation (middle), and (iii) ligand quantification (bottom).

### Analytical Characterization of the IONPs

Prerequisites for our citrate quantification study, summarized in Figure 1, are IONPs with a narrow size distribution, as the dispersity in NP size and shape can lead to enhanced uncertainties for the determination of the NP number concentration and total surface area.^22^ To obtain monodisperse, high-quality, crystalline IONPs with a uniform spherical shape for our multimethod citrate quantification study, we used a thermal decomposition method in apolar solvents, which provides a better monodispersity compared to aqueous syntheses.^34^ The resulting OA-capped IONPs (SI, Figures S1–S3) reveal a uniform spherical shape and a narrow size distribution with size variations of 5%. Subsequently, the hydrophobic OA surface ligands were exchanged for hydrophilic CA. For NMs such as AgNPs, IONPs, quantum dots, and lanthanide NPs, this approach is frequently used for preparing high-quality water-dispersible NMs.^9,35^ As confirmed by TEM, ligand exchange did not affect IONP size and monodispersity (Figure 2, panels A and B). Successful ligand exchange is also supported by the highly negative zeta potential of IONP-CA samples of −40 ± 5 mV under alkaline conditions, while DLS reveals the absence of aggregates (Figure 2, panel B, and SI, Figure S4). However, the desired quantitative exchange of the hydrophobic for the hydrophilic ligands is difficult to achieve and prove. As shown in Figure 2 (panel C), the mass concentration of the IONPs-CA sample obtained gravimetrically, along with the iron oxide (Fe_2_O_3_) concentration calculated from the iron content (5.50 ± 0.05 g/L) determined by ICP-OES (SI, eq 1), indicates a mass not attributable to iron oxide (CA, water residuals, etc.) of 14.6 ± 2.3 wt %. This value provides the upper limit of the citrate content in the sample. From the log-normal fit of the TEM size distribution (SI, eq 2 and 3), an average IONP surface area of 293 nm^2^ and an average IONP volume of 472 nm^3^ were derived. Combining these values with the ICP-OES data and the material density taken from the literature (γ-Fe_2_O_3_; ρ of 4.9 g/cm^3^) gave a particle number concentration and total surface area of the IONPs-CA in the stock dispersion^36^ of 3.40 × 10^15^ particles and 9.96 × 10^17^ nm^2^ per mL (SI, eq 4 and 5).

**Figure 2:**
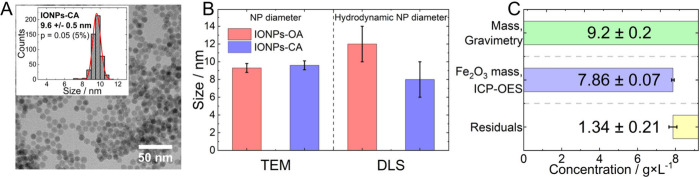
(A) TEM image and size distribution (inset) of the IONPs-CA sample. (B) Bar diagram displaying the size of the IONPs-OA and IONPs-CA samples obtained from TEM images and number-based DLS (error bars refer to the IONP size distribution). (C) Mass concentration of the IONPs-CA sample determined by gravimetry and ICP-OES.

### TGA

The thermogravimetric (TG) curve of sodium citrate dihydrate used as a reference, shown in Figure 3 (red curve), displays four distinct steps of the thermal decomposition (SI, Table S1). The first step corresponds to dehydration^37^ and the other three steps are associated with citrate degradation, in agreement with the literature.^38^ The TG curve of the IONPs-CA sample (Figure 3, blue curve), however, does not show clearly distinguishable steps, but rather multiple mass loss steps. The first step with a mass loss of ∼ 3.5 wt % is ascribed to water elimination. The other steps, associated with a total mass loss of ∼ 12 wt %, are attributed to the degradation of organic species on the IONPs surface. The total mass loss of 15.5 ± 0.3 wt % agrees well with the calculation of the sample composition from a combination of gravimetric and ICP-OES measurements presented above. The observation that the mass loss ratio of IONPs-CA during the three stages of citrate decomposition does not correspond to that of sodium citrate could point to the parallel degradation of other organic compounds, see also the section on RP-HPLC measurements. A more detailed analysis of the TG and differential thermogravimetric (DTG) curves of sodium citrate dihydrate and IONPs-CA can be found in the SI.

**Figure 3:**
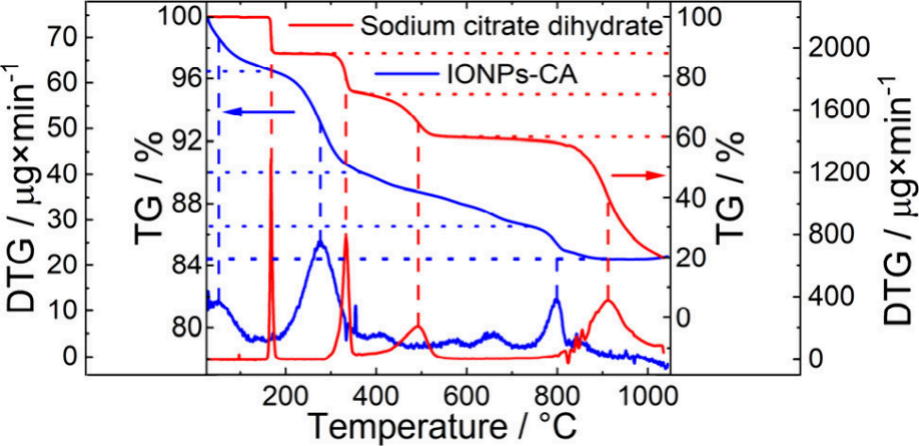
TG and DTG curves of sodium citrate dihydrate (red, right axis) and the IONPs-CA sample (blue, left axis).

### Absorption Spectroscopy

Citrates show a characteristic absorption band at about 210 nm with a small molar absorption coefficient of about 202 M^–1^·cm^–1^, that has been exploited before for citrate quantification.^33^ However, the strong absorption of iron species in the UV/vis interferes with such measurements.^31^ Absorption measurements of IONPs-CA dissolved by addition of HCl (SI, Figure S5) reveal that the contribution from the citrate absorbance in the samples and standards is negligible compared to the high background absorption of the iron species in the wavelength range of 200 to 250 nm. This renders this simple photometric approach not suitable for determining citrate in our IONPs-CA samples.

### RP-HPLC with UV Detection

The challenging high background imposed by the presence of iron ions can be elegantly circumvented by combining a classical separation method such as RP-HPLC, separating different species in solution based on their interaction with the mobile and stationary phases, with optical detection. This is shown in Figure 4 (panel A). CA, corresponding to the peak at about 5.2 min in the HPLC chromatograms, is well separated from chloride and iron species present in solution which pass through a RP-HPLC column without retention (peaks at around 2.5 min), see HPLC chromatograms of all standards in the SI in Figure S6. The calibration curves obtained for the CA standards with and without Fe^3+^ ions (Figure 4, panel B) can be well fitted with a linear regression in the concentration range of 0.01 mM to 0.50 mM using 7 standards per calibration and can be extended to higher CA concentrations. This correlates well with the limit of citrate quantification of 0.016 mM determined by Luo et al.^39^ The calibration curves measured on three different days (SI, Figure S7, panels A-C) confirm a reproducibility with an accuracy of 4%. The presence of iron ions does not affect the citrate peak, which is clearly distinguishable from the background, and hence its quantification from the calibration curves. Fe^3+^ ions do not need to be considered for citrate quantification which was also supported by measurements of the citrate calibration standards containing even higher iron ion concentrations (SI, Figure S7, panel D). The accuracy and precision of citrate quantification with this HPLC approach was confirmed by two control samples A (0.198 mM) and B (0.092 mM) with measured values 0.198 and 0.093 mM, respectively, with measurement error 0.002 mM. Based on our HPLC measurements, the amount of citrate in the as prepared IONPs-CA samples was determined to 0.164 ± 0.002 mM. This corresponds to a citrate concentration of 3.28 ± 0.03 mM in the stock IONPs-CA dispersion and 6.7 ± 0.2 wt % considering the mass concentration of the stock dispersion as determined by drying. This value amounts to only half of the amount of citrate derived from the nonselective TGA method. Apparently, the total organic content determined by TGA cannot solely be attributed to citrate. This suggests the presence of OA ligands on the IONP surface which were not completely replaced by citrate or other organic residuals from the IONP synthesis and/or ligand exchange procedure.

**Figure 4:**
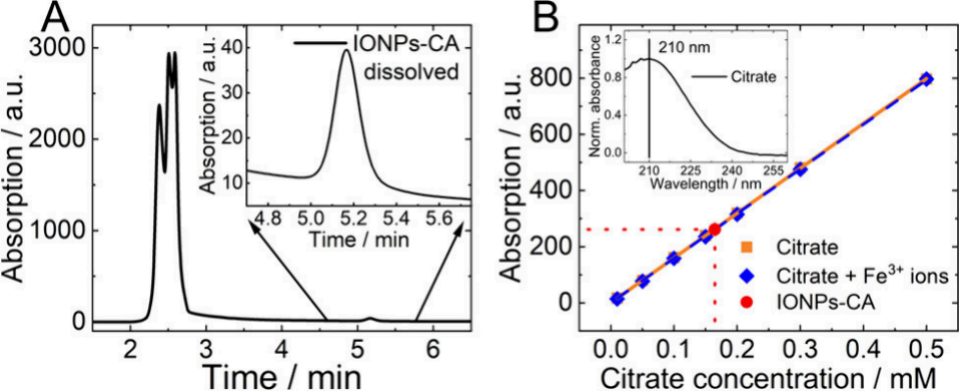
(A) Typical HPLC chromatogram of a dissolved IONPs-CA sample with the inset showing the peak corresponding to citrate; the detection wavelength is 210 nm. (B) Calibration curves used for quantifying the citrate concentration from the HPLC data measured in the absence and presence of Fe^3+^ ions; the inset shows the normalized absorption spectrum of citrate.

### qNMR

Next, we explored solution qNMR for quantifying citrate ligands of dissolved IONPs-CA samples. Thus, in the sample preparation workflow involving drying, weighting, and dissolution with DCl, a step had to be included for the quantitative removal of the released molecular paramagnetic iron species interfering with the qNMR measurements. Iron ions were precipitated by addition of NaOD, forming iron deuteroxide, which was separated from the analyte solution by centrifugation. Thereby, 99.97 ± 0.01% of the iron species were removed, as confirmed by ICP-OES. The NMR spectra of the supernatant obtained for representative IONPs-CA samples are shown in Figure 5 and in the SI (Figure S8). The arbitrarily assigned protons H_A_ and H_B_, which yield two doublet signals due to geminal coupling, were independently integrated within a narrow frequency window to exclude possible influences from impurities. The concentration of citrate in the IONPs-CA stock solution was then calculated by comparing the integrals of the citric acid protons with that of maleic acid (SI, eq 6). This yielded a citrate concentration of 2.95 ± 0.04 mM (6.0 ± 0.1 wt %) in the IONPs-CA stock dispersion. The additional peaks observed in the NMR spectra of the supernatant, e.g., at 8.44, 3.16, and 2.91 ppm, indicate the presence of organic impurities in the sample, originating from IONP synthesis including the reagents used, the ligand exchange process, and the qNMR sample preparation procedure. This finding agrees well with the observed differences between the HPLC and TGA data. Additionally performed NMR spiking experiments revealed that the peaks at 8.44 and 2.28 ppm correspond to formic and acetic acids, respectively, that could possibly be degradation products of CitAc. Based on these qNMR measurements, the concentrations of formic and acetic acids were determined to about 0.49 mM and 0.05 mM, respectively.

**Figure 5:**
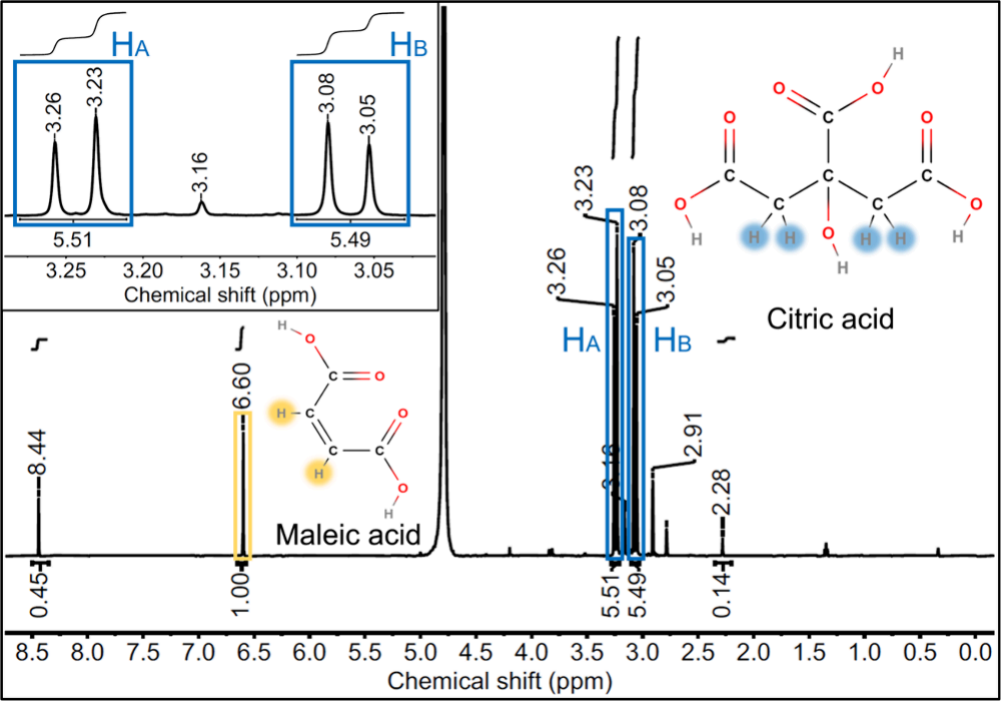
Solution ^1^H NMR spectrum of the supernatant of a dissolved IONPs-CA sample after removal of the iron species, revealing the peak integrals used for citrate quantification by comparison with maleic acid utilized as internal standard (δ = 6.60 ppm). The inset shows the citric acid peaks.

### Elemental Analysis

To better understand the deviations between the TGA, HPLC, and qNMR data, the relative masses of carbon, hydrogen, and nitrogen of the IONPs-CA sample were determined using elemental analysis. With this nonselective method, the citrate amount can be estimated from the measured carbon content, which is, however, also present in other organic compounds such as OA. As detailed in the SI in Table S2, CHN analysis reveals a carbon content of 4.06 ± 0.47 wt %. Considering that one citrate molecule contains six carbon atoms and assuming, that the carbon content solely originates from citrate, this value corresponds to 10.6 ± 1.2 wt % citrate in the IONPs-CA stock dispersion (SI, eq 7). This result matches well with the TGA data. However, 0.34 ± 0.04 wt % nitrogen was also detected in the IONPs-CA sample. These small amounts of nitrogen may originate from DMF used during ligand exchange, as it has been reported that DMF can stabilize IONPs.^40^

### Method Comparison

The citrate concentration obtained from the qNMR data is about 10% lower than the concentration derived from the RP-HPLC measurements. This could point to an underestimation of citrate by qNMR due to loss or degradation of citrate during the additional sample preparation steps required for iron species removal or an overestimation of citrate by RP-HPLC due to a possible overlap of the citrate peak in the HPLC chromatogram with a peak originating from an impurity. To clarify the latter assumption, formic and acetic acids found in the NMR spectra were mixed with citrate and then analyzed by our RP-HPLC method (SI, Figure S9, panel A). Apparently, the peak originating from formic acid does not interfere with the citrate peak, while the acetic acid peak overlaps with the citrate peak. However, the small absorption of acetic acid at the detection wavelength of 210 nm together with its relatively low concentration according to the qNMR measurements (∼0.05 mM) can result in only marginal effects.

Next, to assess a possible influence of the additional sample preparation steps involved in the qNMR workflow, we performed RP-HPLC measurements with supernatant from IONPs-CA samples prepared according to the qNMR sample preparation protocol including iron species removal (SI, Figure S9, panel B). This revealed a decrease in citrate concentration of the sample to about 3.06 mM, equaling a citrate loss of about 7%. Moreover, the RP-HPLC chromatogram contained several small peaks, which could possibly originate from postsynthesis impurities or citrate degradation products. One of these peaks coincides with the formic acid peak. Considering typical measurement uncertainties of qNMR and RP-HPLC methods (including sample preparation, calibration, and measurement steps) of about 5% and 10%, the good match with the qNMR measurements confirms the reliability and accuracy of our HPLC data. This also supports the assumption detailed in the previous section that the organic content of the IONPs-CA sample determined by nonselective TGA and CHN approaches cannot solely be associated with citrate and suggests the presence of minimum amounts of OA or other organic residuals on the IONP surface.

The results obtained by the different citrate quantification methods previously discussed are summarized in Table 1. The citrate density and the number of citrate molecules per IONP can be subsequently calculated from citrate concentration, thereby considering its mode of surface binding (SI, eq 8 and 9). Citrate can bind to the surface of IONPs via one, two, or all three carboxyl groups with the configuration of the citrate molecule on the NP surface influencing its space requirements and footprint. Huang et al.^41^ determined footprints of 0.514, 0.195, and 0.311 nm^2^ for the attachment of citrate to the NP surface using one, two or three carboxyl groups, respectively. The theoretical coverage of a single IONP by a citrate monolayer, calculated for the different modes of citrate surface coordination (SI, eq 10) is presented in the SI in Table S3. The number of citrate molecules per NP calculated based on the citrate concentrations measured by HPLC and qNMR closely agrees with the theoretically calculated value for a citrate monolayer using a footprint of 0.514 nm^2^. This suggests that most citrate molecules are most likely attached to the IONP surface via one carboxyl group.

**Table 1:** Comparison of the Results of Citrate Quantification of IONPs-CA Obtained by the Different Analytical Methods

Parameter	HPLC	qNMR	TGA^a^	Carbon content^a^
Citrate amount [mM]	3.28 ± 0.03	2.95 ± 0.04	5.82 ± 0.53	5.18 ± 0.60
Citrate amount [g/L]	0.62 ± 0.01	0.56 ± 0.01	1.1 ± 0.1	1.0 ± 0.1
Citrate amount [wt%]	6.7 ± 0.2	6.0 ± 0.1	12 ± 1	10.6 ± 1.2
Number of citrate molecules per nm^2^ of IONP surface	1.98 ± 0.02	1.78 ± 0.02	3.52 ± 0.32	3.13 ± 0.36
Number of citrate molecules per IONP	581 ± 5	522 ± 7	1031 ± 94	917 ± 106

a The values were calculated assuming that the organic content of the IONP-CA sample solely originates from citrate.

## Conclusion and Outlook

A multimethod approach for the quantification of citrate (CA), frequently employed as a biocompatible ligand for stabilizing nanoparticles (NPs) in aqueous environments, was presented, employing analytical methods relying on different signal generation and detection principles, as a prerequisite for efficient method cross-validation. As representative and particularly challenging nanomaterial (NM), we chose broadly applied, strongly absorbing, and magnetic iron oxide nanoparticles (IONPs). Analytical methods explored include thermogravimetric (TGA) and elemental (CHN) analysis, which require only particle separation from dispersion and drying for sample preparation and nonselectively provide information on the total amount of the organic content of inorganic NP coatings. Also, simple, and compared to these methods more citrate-selective photometry was assessed, which proved to be unsuitable for the IONP sample. In addition, more advanced and selective reversed-phase high-performance liquid chromatography (RP-HPLC) with absorption (UV) detection and quantitative nuclear magnetic resonance spectroscopy (qNMR) were employed for the first time for ligand analysis on magnetic NPs.

Our results demonstrate that the usage of ligand-nonspecific analytical methods such as TGA and CHN analysis can lead to an overestimation of the ligand amount since a discrimination of signal contributions originating from other substances present in the sample, such as precursors (including impurities) and byproducts from NP synthesis and surface modification as well as solvents residuals, is not feasible. For the IONPs-CA sample, these contributions accounted for up to half of the total organic content measured. Nevertheless, TGA and CHN analysis are well suited to provide a first insight into sample composition and can yield upper limits for other more target-specific methods as shown in this study. For a more realistic picture of the citrate ligand shell and the accurate quantification of citrate surface ligands required to correlate surface chemistry with NM function, citrate selective techniques such as (reversed-phase) HPLC with UV detection and qNMR are needed. To address interferences imposed by IONPs and their constituents for optical and NMR methods, sample preparation workflows were developed for the quantitative separation of absorbing and magnetic iron species and subsequently validated. These workflows can be simply adapted to other similarly challenging NMs.

In summary, our multimethod approach to citrate quantification highlights the advantages of combining specific and unspecific methods for the characterization of NM surface chemistry. This is also important for toxicity studies and NM risk assessment where the potential toxicity of the NM ligand is typically separately assessed. In addition, we could demonstrate the underexplored potential of RP-HPLC with UV detection as an efficient method for quantifying citrate on NPs. Contrary to qNMR, HPLC methods do not require a multistep sample preparation in the case of IONPs and most likely for other NMs as well, which could lead to an enhanced uncertainty. Also, HPLC measurements can be performed with relatively small sample amounts, as follows from the about 20-fold smaller amount of IONPs-CA sample used for the HPLC measurements compared to qNMR. This can be crucial for some applications. We expect that in the future, versatile HPLC methods with photometric, fluorescence and mass spectrometry detection as well as application-specifically adapted workflows for NM sample preparation as derived here for IONPs will receive much more interest for the analysis and quantification of NM surface ligands.

## Supplementary Material



## Notes

Additional experimental details for all characterization methods and the corresponding calculations (PDF)
