# Supplementary material for: Quantifying Citrate Surface Ligands on Iron Oxide Nanoparticles with TGA, CHN Analysis, NMR, and RP-HPLC with UV Detection
Source: Anal Chem. 2025 Aug 29;97(36):19627–34. doi: 10.1021/acs.analchem.5c03024 (PMC12444741; doi:10.1021/acs.analchem.5c03024)
Supplement: Supplementary file 1 [file ac5c03024_si_001.pdf]

Supporting Information (SI)

Quantifying citrate surface ligands on iron oxide nanoparticles with TGA, CHN analysis, NMR, and RP-HPLC with UV detection

Anna Matiushkina[1,2], Sarah-Luise Abram[1], Isabella Tavernaro[1], Robert Richstein[3], Michael R. Reithofer[3], Elina Andresen[1], Matthias Michaelis[1], Matthias Koch[4], and Ute Resch-Genger[1,*]

[1]Division *Biophotonics*, Bundesanstalt für Materialforschung und -prüfung (BAM), Richard-Willstaetter-Straße 11, 12489 Berlin, Germany; email: ute.resch@bam.de

[2]Department of Biology, Chemistry, and Pharmacy, Free University Berlin, Arnimallee 22, 14195 Berlin, Germany

[3]Institute of Inorganic Chemistry, University of Vienna, Waehringer Straße 42, 1090 Vienna, Austria

[4]Division *Organic Trace and Food Analysis*, Bundesanstalt für Materialforschung und -prüfung (BAM), Richard-Willstaetter-Straße 11, 12489 Berlin, Germany

Contents

1. Experimental Information 2
2. Characterization of IONPs 4
   Characterization of oleate (OA) coated IONPs. 4
   Characterization of citrate (CA) coated IONPs. 5
3. Citrate quantification 6
   TGA. 6
   UV-Vis absorption measurements. 7
   RP-HPLC measurements with UV detection. 7
   qNMR measurements. 9
   Elemental (CHN) analysis. 11
   Method comparison. 11
4. References 12

1. Experimental Information

Materials. Iron (III) chloride hexahydrate (≥99%), ethanol absolute (≥99.9%), 1,2-dichlorobenzene (≥98%), N,N-dimethylformamide (≥99.9%), diethyl ether (≥99.5%), glacial acetic acid (≥99.5%), hydrochloric acid (HCl, 37%), methanol (gradient grade for HPLC, ≥99.85%), ethanol (≥99.8%), acetone (≥99%), and cyclohexane (≥99.5%) were obtained from Chemsolute (Th Geyer, Germany). Sodium oleate (≥82%), oleic acid (90%), sodium hydroxide (NaOH, 1N), phosphoric acid (85%), deuterium oxide (D_2O, 99.9%), sodium deuteroxide solution (NaOD, 30% in D_2O), deuterium chloride solution (DCl, 35% in D_2O), maleic acid (standard for qNMR), and the iron standard Titrisol (1000 mg Fe, $FeCl_3$ in 15% HCl) and iron ICP standard traceable to SRM from NIST (1 g/L Fe in 2-3% nitric acid) were purchased from Sigma-Aldrich (Merck, Germany). Sodium acetate trihydrate (pharma grade) and *n*- hexane (≥99%) were obtained from AppliChem GmbH (Germany), 1-octadecene (ODE, 90%) from Thermo Fisher Scientific, sodium citrate tribasic dihydrate (≥99.5%) from Fluka, iron ICP standard from Merck KGaA (Germany), citric acid (99%) from Glentham Life Sciences Ltd. (United Kingdom), and sodium dihydrogen phosphate dihydrate (≥99%) from Carl Roth GmbH (Germany). All chemicals were used without further purification. All aqueous solutions and buffers were prepared with ultrapure water (0.055 $\mu S\cdot m^{-1}$; MilliQ water, Millipore).

Dynamic light scattering (DLS) and zeta potential measurements. DLS and zeta potential measurements were performed at 25 °C with a Zetasizer Nano ZS (Malvern Panalytical Ltd., UK), equipped with a 633 nm laser. For the DLS measurements, a back scattering detection angle of 173° was used. 1 mL of the NP dispersions with concentrations of around 1 g/L were measured in triplicate using 1 cm quartz cuvettes (Hellma GmbH) for oleate-capped iron oxide nanoparticles (IONPs-OA) and 1 cm disposable cuvettes (Sarstedt GmbH) for citrate-capped iron oxide nanoparticles (IONPs-CA). The hydrodynamic diameter was derived with the cumulant method and calculated for a number-based distribution. Zeta potential measurements were done in triplicate with disposable capillary cells (Malvern Panalytical, DTS1070) for IONPs-CA dispersion with a concentration of around 2 g/L in water at a pH of 9.2. The zeta potential was determined based on the electrophoretic mobility of the nanoparticles using the Smoluchowski model. All DLS and zeta potential measurements were analyzed using refractive indexes of 2.440, 1.330, and 1.427 for iron oxide, water, and cyclohexane, respectively, as well as viscosities of 0.8872 cP and 1.0000 cP for water and cyclohexane.

Transmission electron microscopy (TEM). TEM measurements were carried out using a Talos F200S microscope (Thermo Fisher Scientific) with an accelerating voltage of the electron beam of 200 kV. The samples were prepared on carbon-coated copper grids (Plano GmbH) by drop casting and drying at r.t. The obtained TEM micrographs were analyzed using the software ImageJ (Version 1.54g), evaluating 300 and 500 particles for IONPs-OA and IONPs-CA, respectively.

Determination of the IONP iron content. The iron content of the IONPs was determined by inductively coupled plasma optical emission spectrometry (ICP-OES) using a SPECTRO Arcos-EOP (Model: FHX, 76004553) spectrometer (SPECTRO Analytical Instruments). For instrument calibration, iron ion standard solutions with concentrations of 0.025 - 0.500 mg/L prepared from an iron ICP standard solution (SRM from NIST, Sigma-Aldrich). The IONPs-CA samples were dissolved with HCl (37%) and further diluted in MilliQ water prior to the ICP-OES measurements.

Thermogravimetric analysis (TGA). TGA was performed using a Hitachi STA 7200 set-up with an AS3 Sample Charger. Thermogravimetric (TG) curves and the corresponding derivative thermogravimetric (DTG) curves were recorded under argon atmosphere at

heating rates of 10 °C/min for IONPs-CA and 20 °C/min for IONPs-OA and sodium citrate dihydrate.

Elemental (CHN) analysis. Elemental analyses were performed at the 'Service d'analyse' of LCC using a PerkinElmer 2400 Series II analyzer. Approximately 1 mg of two independent samples of dried IONPs-CA were accurately weighed and then dried under vacuum at 105 °C in air. Each sample was measured in duplicates to ensure reproducibility, and the average value was used for further analysis.

Reversed-phase high-performance liquid chromatography (RP-HPLC) with UV detection. HPLC experiments were performed using a 1260 Infinity system from Agilent Technologies equipped with a diode array detector (DAD). An Eurospher II 100-5 C18 (250 x 4.6 mm) column was used, the injection volume was 20 µL, the flow rate 1 mL/min, and the column temperature 30 °C. The mobile phase consisted of A: a phosphate buffer (pH 2.9 ± 0.1; 0.5% sodium dihydrogen phosphate solution, pH adjusted with 85% phosphoric acid) and B: methanol in an isocratic ratio of A:B = 97.5:2.5. The signal was recorded at 210 nm at the absorption maximum of citric acid.

Three independent samples were prepared by dissolving 50 µL of IONPs-CA stock dispersion with 200 µL of HCl (37%) and subsequent dilution with 750 µL of MilliQ water. Each sample was measured three times, and the experiment was repeated twice. The citrate standards with citrate concentrations of 0.01 - 0.50 mM required for quantification were prepared by dissolving sodium citrate tribasic dihydrate in MilliQ water, followed by a dilution step and addition of a same amount of HCl (37%) as used for the dissolution of IONPs-CA. Additionally, to consider a possible impact of Fe^{3+} ions released from the dissolved IONPs, citrate standards containing Fe^{3+} ions at the same concentration as the IONPs-CA samples (0.276 g/L) were prepared by adding the respective amount of Fe^{3+} from the Titrisol iron standard. All HPLC standards and samples had a pH of 0.

Absorption spectroscopy. Photometric measurements were carried out with a Cary 5000 spectrophotometer (Agilent Technologies). Therefore, the samples and standards prepared for the HPLC measurements were diluted twice with a dilute HCl solution (7.4%), yielding calibration standards with citrate concentrations of 0.005 - 0.250 mM without and with Fe^{3+} ions (0.138 g/L). For the photometric measurements, 2 mm quartz cuvettes were used and either the respective solvent, i.e., a diluted HCl solution with and without Fe^{3+} ions as references.

Solution quantitative nuclear magnetic resonance (qNMR) spectroscopy. The qNMR spectra were recorded at 25 °C using a Bruker BioSpin AV III 600 spectrometer operating at 600.25 MHz for ^1H detection. Each spectrum was acquired using 64 scans with a calibrated 90° excitation pulse (acquisition time 2.6 s) and a 45-second pulse delay (~7 times the relaxation time of maleic acid). Spectral processing involved the use of efp with a line broadening parameter (lb = 0.3 Hz). Chemical shifts (δ) are reported in ppm, with the residual protic solvent peak from water (D_2O: δ ^1H = 4.79 ppm) used as a reference. The spectra were recorded over a range of -4 to 16 ppm and processed using TopSpin (Version 4.0.8) software, with subsequent analysis performed using MestreNova (Version 15.0.1) software. Prior to integration, each spectrum underwent phase and baseline correction. For quantitative analysis, manual integration of the maleic acid signal (6.57 - 6.65 ppm, 2H) was performed and the integral was compared with the integrals of the citric acid signals (3.03 - 3.10 ppm, 2H; 3.21 - 3.28 ppm, 2H) to determine the citrate concentration.

For the qNMR measurements, three independent samples were prepared. Therefore, 1 mL of a IONPs-CA dispersion were dried, the particle mass was determined gravimetrically, and the particles were dissolved in a DCl (35%):D_2O mixture (150:150 µL) at r.t. After around 1 h, 200 µL of a 30% NaOD solution with 500 µL of D_2O were added yielding iron deuteroxide and the samples were shaken at 50 °C for 3 d. Then, iron deuteroxide was separated by

centrifugation. For the qNMR measurements, 800 µL of the supernatant were taken and 25 µL of a maleic acid solution (2 g/L, prepared using a Sartorius Cubis MCM 6.7 balance) were added as an internal standard. The pH was adjusted to 2-3 with DCl.

2. Characterization of IONPs

Characterization of oleate (OA) coated IONPs.

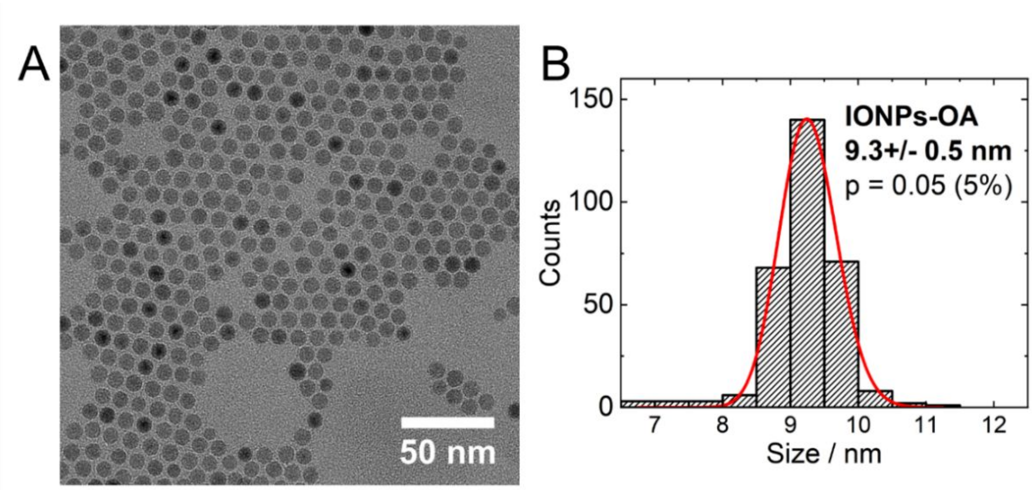

Figure S1. TEM image (A) and size distribution (B) of IONPs-OA.

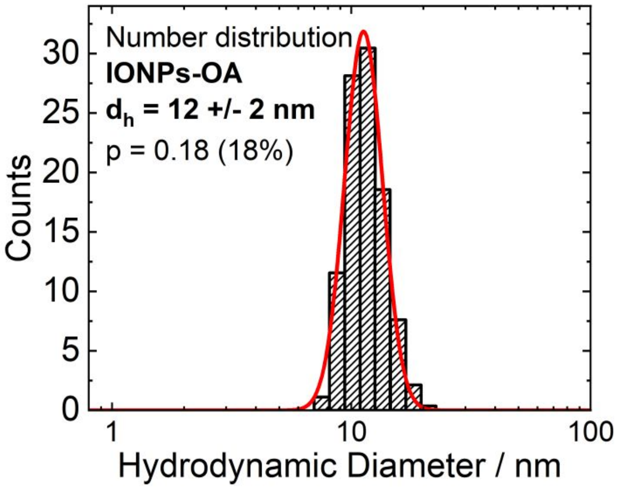

Figure S2. Number-based size distribution of IONPs-OA in cyclohexane as obtained by DLS measurements.

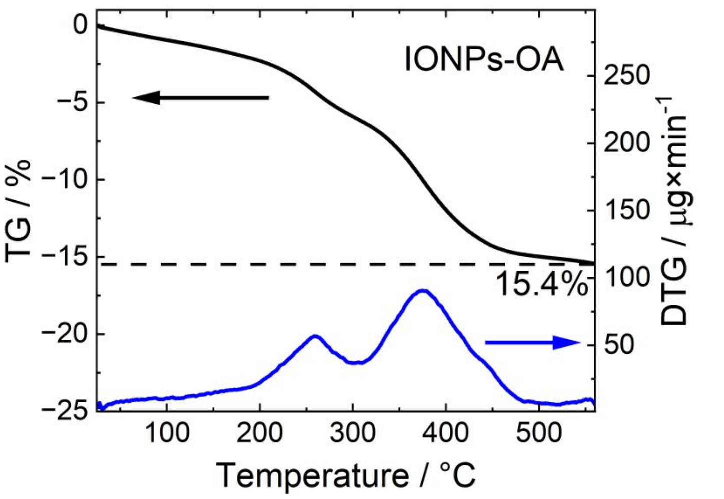

Figure S3. TG and DTG curves of dried IONPs-OA.

Characterization of citrate (CA) coated IONPs.

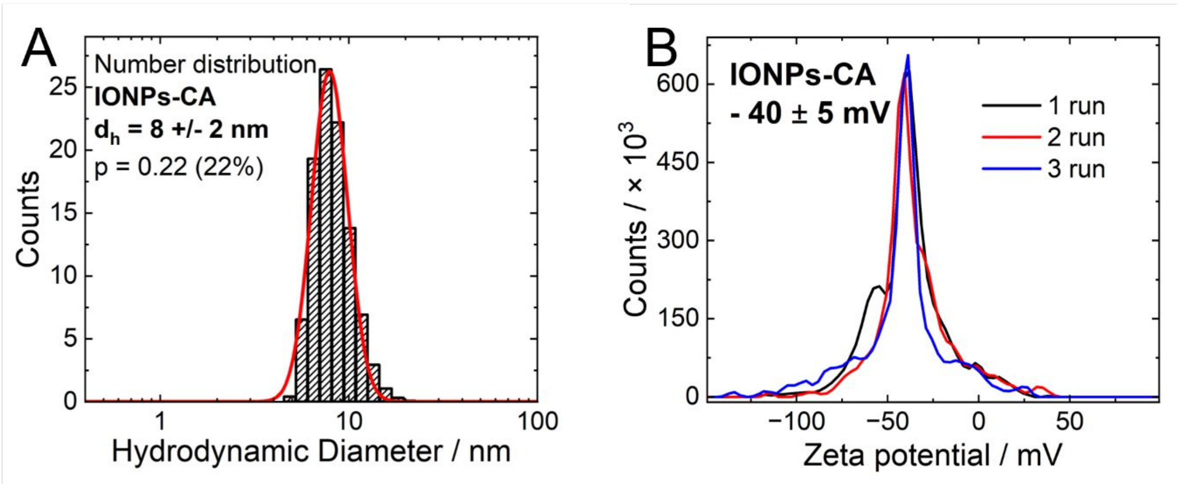

Figure S4. Number-based size distribution obtained by DLS (A) and zeta potential curves (B) of IONPs-CA dispersed in water (pH of 9.2).

The iron oxide mass concentration ($C_m(Fe_2O_3)$) was calculated via:

$$C_m(Fe_2O_3) = \frac{C_m(Fe)}{M(Fe)} \cdot \left(M(Fe) + \frac{3}{2} \cdot M(O)\right) = = \frac{5.50\ g/L}{55.845\ g/mol} \cdot \left(55.845 \frac{g}{mol} + \frac{3}{2} \cdot \frac{15.999g}{mol}\right) \approx 7.86\ g/L \quad (1)$$

where $C_m(Fe)$ is the iron mass concentration, and $M(Fe)$ and $M(O)$ are the molar masses of iron and oxygen, respectively.

The average IONP surface area ($\bar{S}_{IONP}$) and the average IONP volume ($\bar{V}_{IONP}$) were calculated following:

$$\bar{S}_{IONP} = \int \frac{F(x)}{\int F(x)dx} \cdot 4\pi \cdot \left(\frac{x}{2}\right)^2 dx = 293\ nm^2 \quad (2)$$

$$\bar{V}_{IONP} = \int \frac{F(x)}{\int F(x)dx} \cdot \frac{4\pi}{3} \cdot \left(\frac{x}{2}\right)^3 dx = 472\ nm^3 \quad (3)$$

where $F(x)$ is the log-normal fit of the IONPs-CA size distribution derived from TEM images.

The estimated number of IONPs-CA (N_{IONPs}) and their total surface area (S_{IONPs}) in 1 mL of the stock dispersion were calculated according to:

$$N_{IONPs} = \frac{C_m(Fe_2O_3) \cdot V_{solution}}{m_{IONP}} = \frac{C_m(Fe_2O_3) \cdot V_{solution}}{\overline{V}_{IONP} \cdot \rho(Fe_2O_3)} =$$

$$= \frac{7.86\ g/L \cdot 1\ mL}{472\ nm^3 \cdot 4.9\ g/cm^3} \approx 3.40 \cdot 10^{15} \quad (4)$$

$$S_{IONPs} = N_{IONPs} \cdot \overline{S}_{IONP} = 3.40 \cdot 10^{15} \cdot 293\ nm^2 \approx 9.96 \cdot 10^{17}\ nm^2 \quad (5)$$

where $V_{solution}$ is the solution volume, m_{IONP} is the mass of one IONP, and $\rho(Fe_2O_3)$ the density of maghemite, i.e., Fe_2O_3, respectively.

3. Citrate quantification

TGA.

The mass losses and temperatures (derived from the DTG curve) corresponding to each step are presented in Table S1. The first step of the sodium citrate dihydrate thermal decomposition with a mass loss of about 12%, corresponding to its dehydration,[1] occurs at 167 °C. The other three stages at about 333 °C, 492 °C, and 911 °C are associated with the citrate degradation in good agreement with the literature.[2] We assume that the second and third steps correspond to the loss of CO_2 from the carboxyl groups of citrate, while the last step relates to the loss of CO_2 and acetone.[3] The calculated mass losses for these steps are in good agreement with the obtained results.

Table S1. TGA data of sodium citrate dihydrate.

Step	Temperature, °C	Mass loss, %	Released species	Theoretical calculation, %
1	167	~12	dehydration	12.2
2	333	~14	CO_2	15.0
3	492	~14	CO_2	15.0
4	911	~40	CO_2 + acetone	34.4
Residuals	-	~20	3Na	23.4

The second and fourth stages of the thermal decomposition of IONPs-CA correspond to temperatures of 276 °C and 798 °C in the DTG curve. However, the third stage is very smooth and does not have a corresponding maximum in the DTG curve unlike the sodium citrate dihydrate degradation process. The shift of the degradation steps to lower temperatures has been previously observed for IONPs coated with citrate and could be related to a catalytic behaviour of IONPs.[1]

UV-Vis absorption measurements.

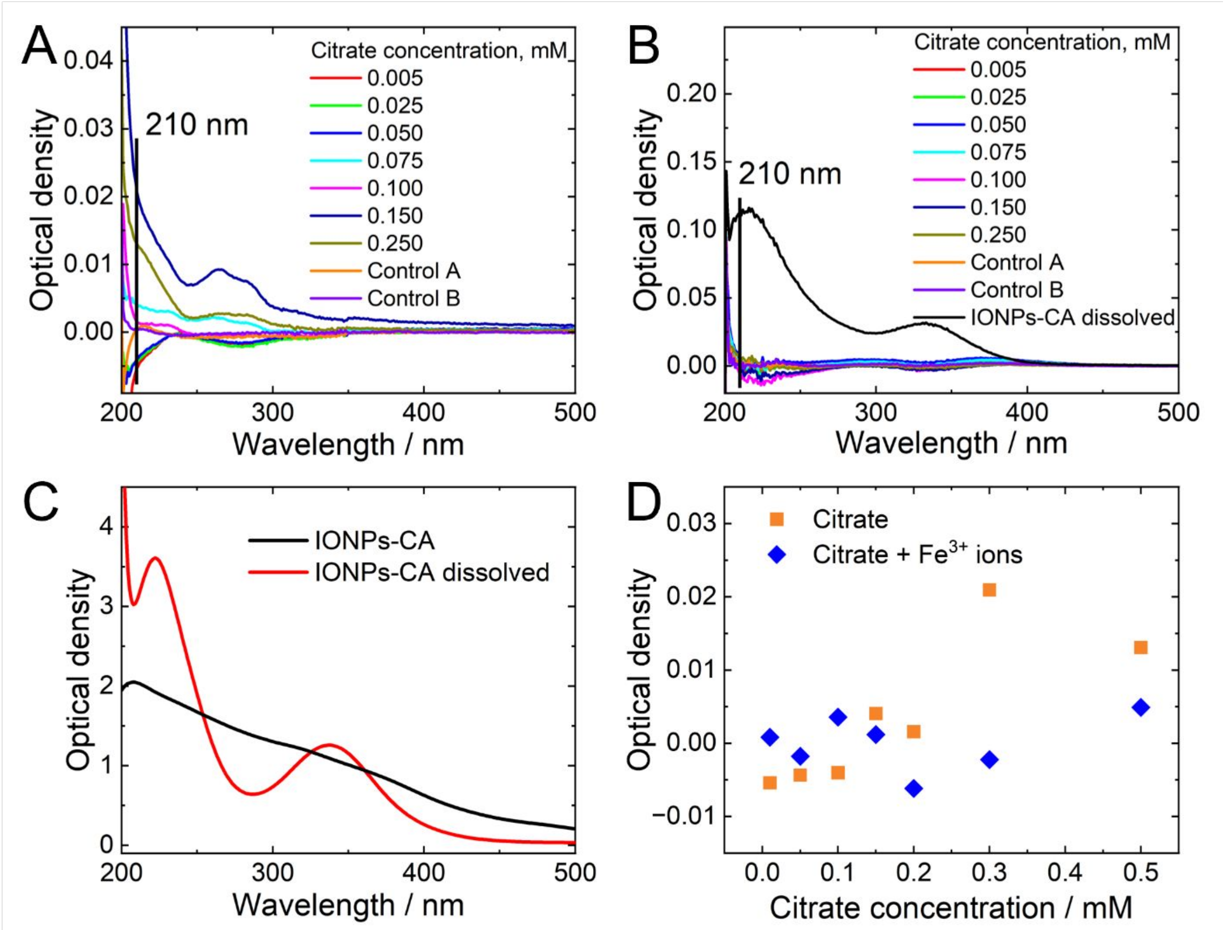

Figure S5. Optical density spectra of the citrate standard solutions (A) without and (B) with addition of Fe^{3+} ions after blank correction (subtraction of the solvent spectrum); all solutions measured had a pH of 0. (C) Typical optical density spectra of IONPs-CA before (pH 9.8) and after IONP dissolution (pH 0) with HCl. (D) Dependence of the absorbance at 210 nm on citrate concentration for the standards used for the photometric measurements.

RP-HPLC measurements with UV detection.

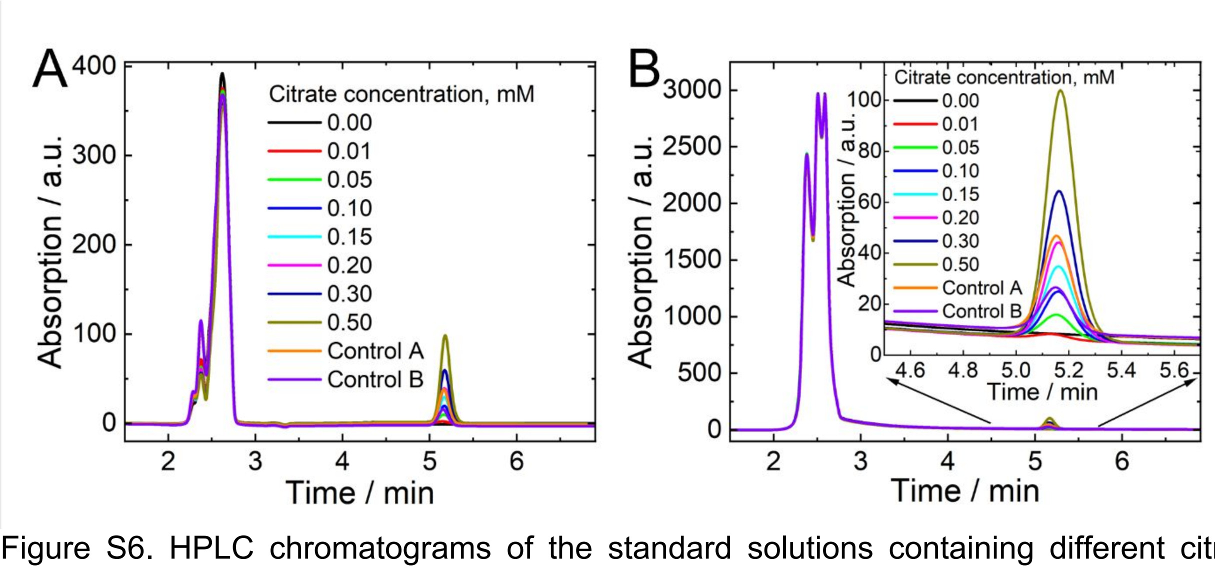

Figure S6. HPLC chromatograms of the standard solutions containing different citrate concentrations (A) without and (B) with iron ions; the inset shows the peak corresponding to citrate.

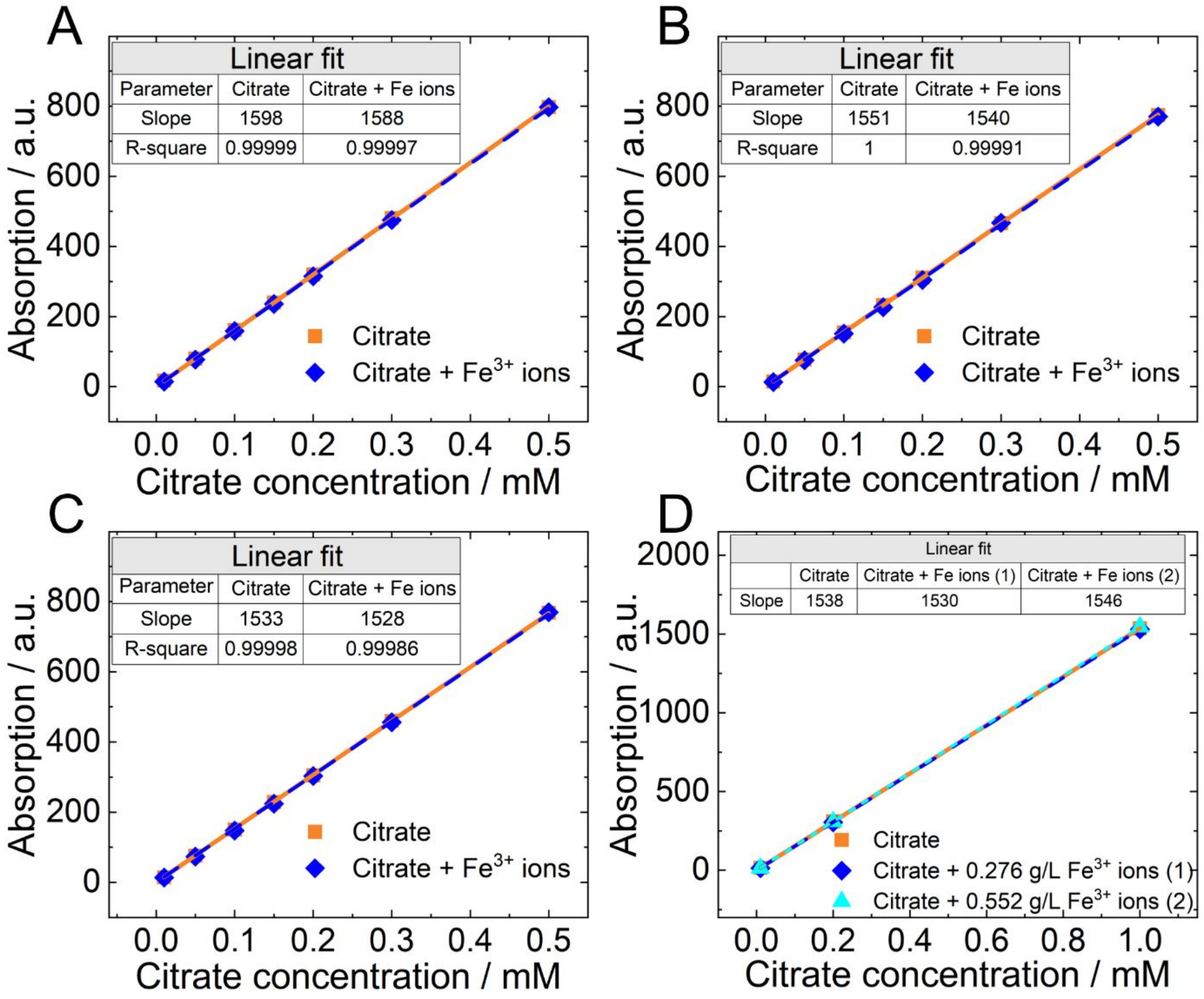

Figure S7. (A-C) Citrate calibration curves used for citrate quantification from the HPLC data obtained on three different days. (D) Calibration curves derived from the HPLC data obtained for citrate standard solutions containing different concentrations of iron ions.

qNMR measurements.

Maleic acid, used as an internal standard for the qNMR measurements, shows one peak at around 6.60 ppm corresponding to the two vinylic hydrogen atoms. The analyte citric acid is characterized by two doublets which arise from geminal coupling (H_A, H_B *J = 16.1 Hz*) in the range of 3.01 - 3.30 ppm. For the quantitative analysis of the NMR spectra, the doublets H_A and H_B (both representing two hydrogen atoms) were separately integrated to exclude the contribution of an impurity peak that was visible at around 3.16 ppm. The integration windows of the doublets H_A and H_B are chosen to be narrow to avoid interferences with impurities. Since the proton proportion of maleic acid and H_A and H_B doublets of citric acid is 1:1, the values are not changed in the spectra.

The calculation of the citrate concentration ($C(citrate)$) in the IONPs-CA stock dispersion was performed via:

$$C(citrate) = \frac{\nu(maleic\ acid) \cdot I(citric\ acid)}{V(qNMR)} = \frac{0.429\ \mu mol \cdot 5.51}{0.8\ mL} = 2.95\ mM \qquad (6)$$

where $\nu(maleic\ acid)$ is the maleic acid amount, $I(citric\ acid)$ is the integration value of citric acid (the integration value of maleic acid is taken as 1), and $V(qNMR)$ is the volume of IONPs-CA sample used for qNMR.

The concentrations of formic and acetic acids were calculated in the same way, thereby taking into account that the formic acid peak at 8.44 ppm represents one hydrogen atom and the acetic acid peak at 2.28 ppm corresponds to three hydrogen atoms.

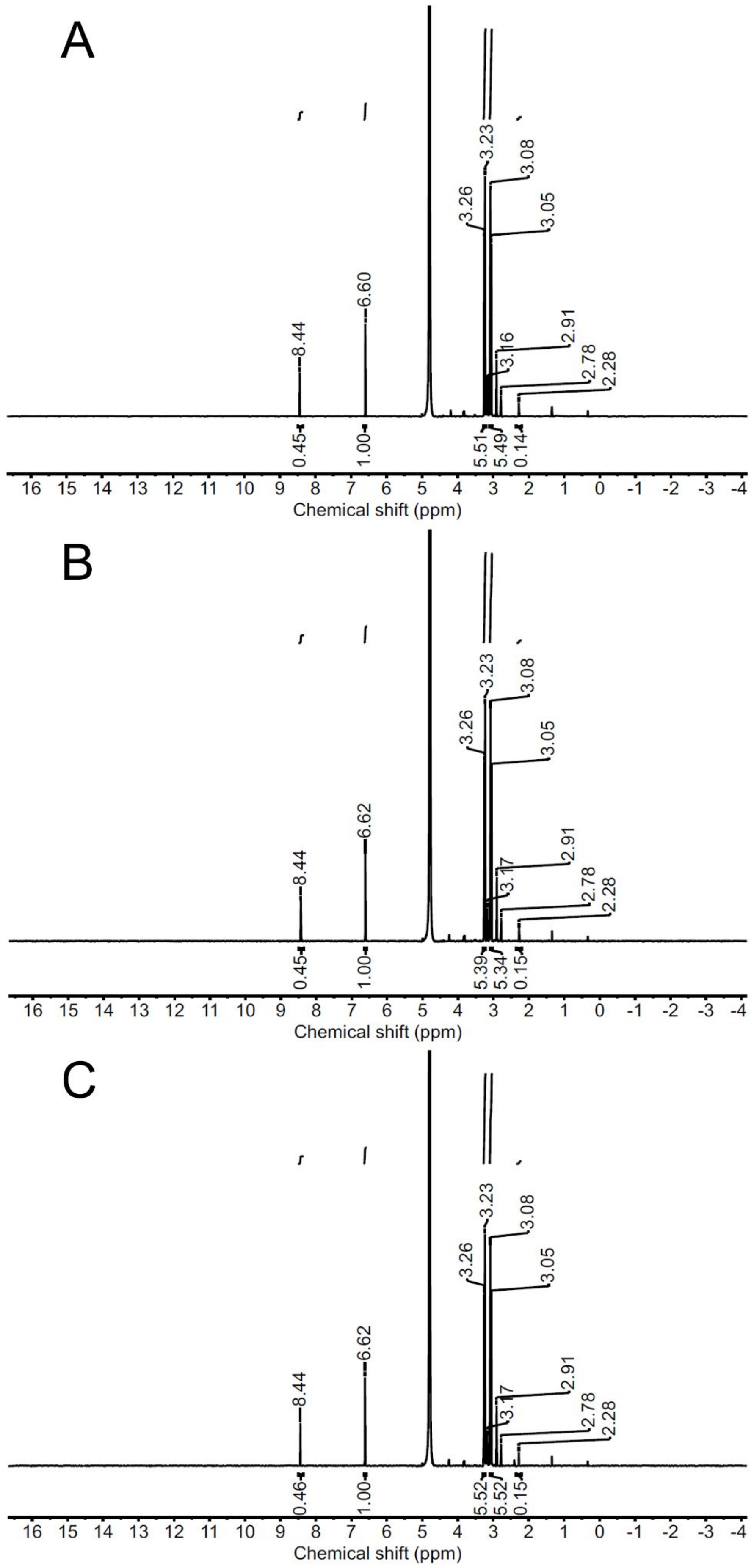

Figure S8. (A-C) Solution ^{1}H NMR spectra obtained after the removal of the iron species. All spectra of the triplicate measurements are shown.

Elemental (CHN) analysis.

Table S2. Mass content of IONPs-CA based on CHN analysis.

**Element**	**Mass, wt%**
Carbon	4.06 ± 0.47
Hydrogen	0.57 ± 0.04
Nitrogen	0.34 ± 0.04

Calculation of the amount of citrate ($C_m(citrate)$) based on the determined content of carbon:

$$C_m(citrate) = C_m(carbon) \cdot \frac{M(citrate)}{6 \cdot M(carbon)} =$$

$$= 4.06\, wt\% \cdot \frac{189\ g/mol}{6 \cdot 12.011\ g/mol} \approx 10.6\, wt\% \quad (7)$$

Method comparison.

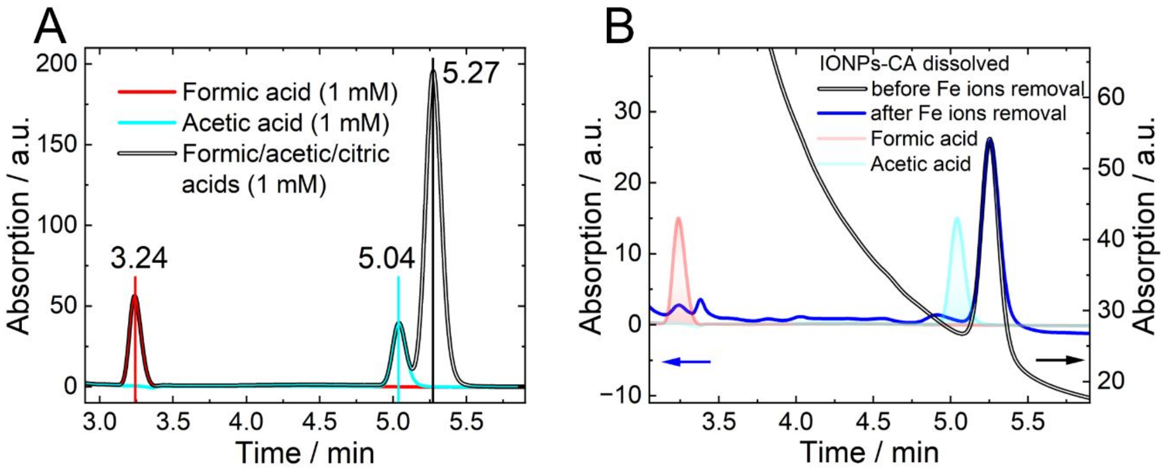

Figure S9. (A) HPLC chromatograms of formic, acetic, and citric acids; all acid concentrations are 1 mM. (B) HPLC chromatograms of dissolved IONPs-CA sample before (right axis) and after (left axis) iron species removal, and normalized HPLC chromatograms of formic and acetic acids.

The number of citrate molecules per nm^2 of NP surface area ($N_{citrate}$) and per one IONP particle ($N'_{citrate}$) derived from, e.g., the HPLC results can be calculated according to:

$$N_{citrate} = \frac{C_{citrate} \cdot V_{solution} \cdot N_A}{S_{IONPs}} = \frac{3.28\ mM \cdot 1\ mL \cdot 6.022 \cdot 10^{23}\ mol^{-1}}{9.96 \cdot 10^{17}} \approx 1.98 \quad (8)$$

$$N'_{citrate} = N_{citrate} \cdot \bar{S}_{IONP} \approx 1.98 \cdot 293 \approx 581 \quad (9)$$

where $C_{citrate}$ is a citrate concentration and N_A is the Avogadro constant.

The number of citrate molecules per nm^2 of surface area ($N_{citrate}$) based on the citrate footprint ($S_{citrate}$) can be calculated according to:

$$N_{citrate} = \frac{1}{S_{citrate}} = \frac{1}{0.514} \approx 1.95 \quad (10)$$

Table S3. Theoretical calculation of citrate coverage.

**Parameter**	**Number of citrate carboxyl groups involved in the attachment to the IONP surface**		
	1	2	3
Citrate molecule footprint,[4] nm^2	0.514	0.195	0.311
Number of citrate molecules per nm^2 of IONP surface	1.95	5.13	3.22
Number of citrate molecules per IONP	570	1503	942

4. References

(1) Saraswathy, A.; Nazeer, S. S.; Jeevan, M.; Nimi, N.; Arumugam, S.; Harikrishnan, V. S.; Varma, P. R. H.; Jayasree, R. S. Citrate coated iron oxide nanoparticles with enhanced relaxivity for in vivo magnetic resonance imaging of liver fibrosis. *Colloid Surface B* **2014**, *117*, 216-224. DOI: https://doi.org/10.1016/j.colsurfb.2014.02.034.

(2) Marcilla, A.; Gómez-Siurana, A.; Beltrán, M.; Martínez-Castellanos, I.; Blasco, I.; Berenguer, D. TGA-FTIR study of the pyrolysis of sodium citrate and its effect on the pyrolysis of tobacco and tobacco/SBA-15 mixtures under N_2 and air atmospheres. *J Sci Food Agr* **2018**, *98* (15), 5916-5931. DOI: https://doi.org/10.1002/jsfa.9121.

(3) Granath, T.; Mandel, K.; Löbmann, P. The Significant Influence of the pH Value on Citrate Coordination upon Modification of Superparamagnetic Iron Oxide Nanoparticles. *Part Part Syst Char* **2022**, *39* (3). DOI: https://doi.org/10.1002/ppsc.202100279.

(4) Huang, T.; Nallathamby, P. D.; Xu, X. H. N. Photostable Single-Molecule Nanoparticle Optical Biosensors for Real-Time Sensing of Single Cytokine Molecules and Their Binding Reactions. *J Am Chem Soc* **2008**, *130* (50), 17095-17105. DOI: https://doi.org/10.1021/ja8068853. Sasidharan, S.; Jayasree, A.; Fazal, S.; Koyakutty, M.; Nair, S. V.; Menon, D. Ambient temperature synthesis of citrate stabilized and biofunctionalized, fluorescent calcium fluoride nanocrystals for targeted labeling of cancer cells. *Biomater Sci-Uk* **2013**, *1* (3), 294-305. DOI: https://doi.org/10.1039/C2BM00127F.
